# Diaqua­bis[4-(dimethyl­amino)benzoato](isonicotinamide)zinc(II)

**DOI:** 10.1107/S1600536809017620

**Published:** 2009-05-14

**Authors:** Tuncer Hökelek, Hakan Dal, Barış Tercan, Özgür Aybirdi, Hacali Necefoğlu

**Affiliations:** a Hacettepe University, Department of Physics, 06800 Beytepe, Ankara, Turkey; b Anadolu University, Faculty of Science, Department of Chemistry, 26470 Yenibağlar, Eskişehir, Turkey; c Karabük University, Department of Physics, 78050, Karabük, Turkey; d Kafkas University, Department of Chemistry, 63100 Kars, Turkey

## Abstract

The mol­ecule of the title Zn^II^ complex, [Zn(C_9_H_10_NO_2_)_2_(C_6_H_6_N_2_O)(H_2_O)_2_], contains two 4-(dimethyl­amino)benzoate (DMAB) ligands, one isonicotinamide (INA) ligand and two water mol­ecules; one of the DMAB ions acts as a bidentate ligand while the other and INA are monodentate ligands. The four O atoms in the equatorial plane around the Zn atom form a distorted square-planar arrangement, while the distorted octa­hedral coordination is completed by the N atom of the INA ligand and the O atom of the water mol­ecule in the axial positions. Intra­molecular C—H⋯O hydrogen bonding results in the formation of a six-membered ring adopting an envelope conformation. The dihedral angle between the carboxyl groups and the adjacent benzene rings are 4.87 (16) and 2.2 (2)°, while the two benzene rings are oriented at a dihedral angle of 65.13 (8)°. The dihedral angle between the benzene and pyridine rings are 11.47 (7) and 74.83 (8)°, respectively. In the crystal structure, inter­molecular O—H⋯O, O—H⋯N and N—H⋯O hydrogen bonds link the mol­ecules into a supra­molecular structure. π–π contacts between the pyridine and benzene rings and between the benzene rings [centroid–centroid distances = 3.695 (1) and 3.841 (1) Å, respectively] further stabilize the structure. Weak inter­molecular C—H⋯π inter­actions are also present.

## Related literature

For general backgroud, see: Antolini *et al.* (1982 ▶); Chen & Chen (2002 ▶); Amiraslanov *et al.* (1979 ▶); Bigoli *et al.* (1972 ▶); Hauptmann *et al.* (2000 ▶); Shnulin *et al.* (1981 ▶); Antsyshkina *et al.* (1980 ▶); Adiwidjaja *et al.* (1978 ▶); Catterick *et al.* (1974 ▶); Krishnamachari (1974 ▶). For related structures, see: Greenaway *et al.* (1984 ▶); Hökelek *et al.* (1995 ▶, 1997 ▶, 2007 ▶, 2008 ▶); Hökelek & Necefoğlu (1996 ▶, 1997 ▶, 2007 ▶).
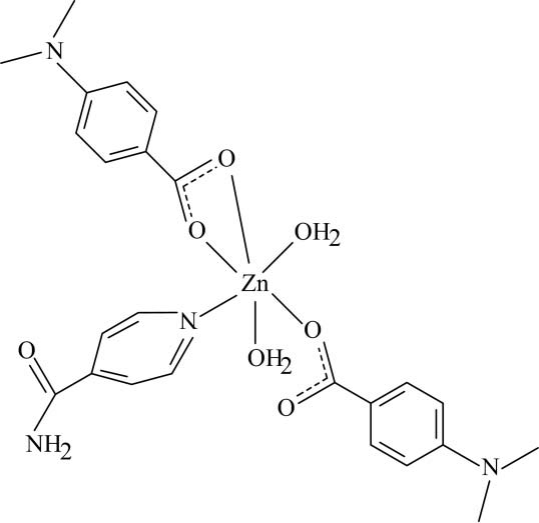


## Experimental

### 

#### Crystal data

[Zn(C_9_H_10_NO_2_)_2_(C_6_H_6_N_2_O)(H_2_O)_2_]
*M* *_r_* = 551.91
Triclinic, 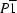

*a* = 6.8616 (2) Å
*b* = 8.0947 (3) Å
*c* = 22.4953 (4) Å
α = 90.683 (2)°
β = 92.838 (2)°
γ = 93.313 (3)°
*V* = 1245.69 (6) Å^3^
*Z* = 2
Mo *K*α radiation
μ = 1.04 mm^−1^
*T* = 100 K
0.57 × 0.27 × 0.20 mm

#### Data collection

Bruker Kappa APEXII CCD area-detector diffractometer
Absorption correction: multi-scan (*SADABS*; Bruker, 2005 ▶) *T*_min_ = 0.720, *T*_max_ = 0.810
21315 measured reflections
6053 independent reflections
5647 reflections with *I* > 2σ(*I*)
*R*_int_ = 0.021

#### Refinement

*R*[*F* ^2^ > 2σ(*F* ^2^)] = 0.040
*wR*(*F* ^2^) = 0.078
*S* = 1.29
6053 reflections
353 parameters
6 restraints
H atoms treated by a mixture of independent and constrained refinement
Δρ_max_ = 0.43 e Å^−3^
Δρ_min_ = −0.99 e Å^−3^

### 

Data collection: *APEX2* (Bruker, 2007 ▶); cell refinement: *SAINT* (Bruker, 2007 ▶); data reduction: *SAINT*; program(s) used to solve structure: *SHELXS97* (Sheldrick, 2008 ▶); program(s) used to refine structure: *SHELXL97* (Sheldrick, 2008 ▶); molecular graphics: *ORTEP-3 for Windows* (Farrugia, 1997 ▶); software used to prepare material for publication: *WinGX* (Farrugia, 1999 ▶) and *PLATON* (Spek, 2009 ▶).

## Supplementary Material

Crystal structure: contains datablocks I, global. DOI: 10.1107/S1600536809017620/xu2522sup1.cif

Structure factors: contains datablocks I. DOI: 10.1107/S1600536809017620/xu2522Isup2.hkl

Additional supplementary materials:  crystallographic information; 3D view; checkCIF report

## Figures and Tables

**Table 1:** Selected bond lengths (Å)

Zn1—O1	2.0353 (17)
Zn1—O3	2.1328 (17)
Zn1—O4	2.2584 (17)
Zn1—O6	2.1393 (17)
Zn1—O7	2.0647 (17)
Zn1—N1	2.1307 (19)

**Table 2:** Hydrogen-bond geometry (Å, °)

*D*—H⋯*A*	*D*—H	H⋯*A*	*D*⋯*A*	*D*—H⋯*A*
N2—H21⋯O4^i^	0.89 (3)	2.15 (3)	3.034 (3)	174 (3)
N2—H22⋯O3^ii^	0.89 (3)	1.95 (3)	2.820 (3)	164 (3)
O6—H61⋯O2	0.89 (3)	1.79 (3)	2.646 (2)	161 (3)
O6—H62⋯O5^iii^	0.88 (3)	1.88 (3)	2.749 (2)	169 (3)
O7—H71⋯N3^iv^	0.89 (3)	2.01 (3)	2.863 (3)	162 (3)
O7—H72⋯O2^v^	0.87 (3)	1.80 (3)	2.667 (2)	171 (3)
C22—H22*A*⋯*Cg*3^vi^	0.96	2.74	3.567 (3)	145
C23—H23*C*⋯*Cg*2^vii^	0.96	2.86	3.644 (3)	140

Symmetry codes: (i) 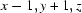; (ii) 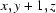; (iii) 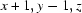; (iv) 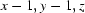; (v) 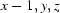; (vi) 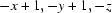; (vii) 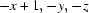. *Cg*2 and *Cg*3 are the centroids of the C9–C14 and N1/C15–C19 rings, respectively.

**Fig. 1.**
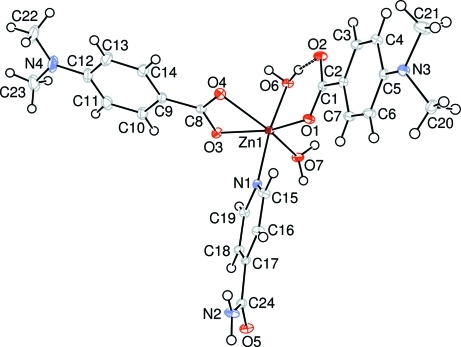
The molecular structure of the title molecule with the atom-numbering scheme. Displacement ellipsoids are drawn at the 50% probability level. Hydrogen bond is shown as dashed line.

## supplementary crystallographic information

### Comment

Nicotinamide (NA) is one form of niacin. A deficiency of this vitamin leads to loss of copper from the body, known as pellagra disease. Victims of pellagra show unusually high serum and urinary copper levels (Krishnamachari, 1974). The nicotinic acid derivative *N*,*N*-Diethylnicotinamide (DENA) is an important respiratory stimulant (Bigoli *et al.*, 1972). Transition metal complexes with biochemical molecules show interesting physical and/or chemical properties, through which they may find applications in biological systems (Antolini *et al.*, 1982). Some benzoic acid derivatives, such as 4-aminobenzoic acid, have been extensively reported in coordination chemistry, as bifunctional organic ligands, due to the varieties of their coordination modes (Chen & Chen, 2002; Amiraslanov *et al.*, 1979; Hauptmann *et al.*, 2000).

The structure-function-coordination relationships of the arylcarboxylate ion in Zn^II^ complexes of benzoic acid derivatives may also change depending on the nature and position of the substituted groups on the benzene ring, the nature of the additional ligand molecule or solvent, and the pH and temperature of synthesis (Shnulin *et al.*, 1981; Antsyshkina *et al.*, 1980; Adiwidjaja *et al.*, 1978). When pyridine and its derivatives are used instead of water molecules, the structure is completely different (Catterick *et al.*, 1974).

The structure determination of the title compound, (I), a zinc complex with two 4-dimethylaminobenzoate (DMAB) and one isonicotinamide (INA) ligands and two water molecules, was undertaken in order to determine the properties of the ligands and also to compare the results obtained with those reported previously.

In the monomeric title complex, (I), the Zn atom is surrounded by two DMAB and INA ligands and two water molecules. One of the DMAB ions acts as a bidentate ligand, while the other and INA are monodentate ligands (Fig. 1). The four O atoms (O1, O3, O4 and O7 atoms) in the equatorial plane around the Zn atom form a highly distorted square-planar arrangement, while the distorted octahedral coordination is completed by the N atom of the INA ligand (N1) and the O atom of the water molecule (O6) in the axial positions (Table 1 and Fig. 1).

The near equality of the C1—O1 [1.265 (3) Å], C1—O2 [1.265 (3) Å], C8—O3 [1.278 (3) Å] and C8—O4 [1.271 (3) Å], bonds in the carboxylate group indicates a delocalized bonding arrangement, rather than localized single and double bonds, and may be compared with the corresponding distances: 1.256 (6) and 1.245 (6) Å in [Mn(DENA)_2_(C_7_H_4_ClO_2_)_2_(H_2_O)_2_], (II) (Hökelek *et al.*, 2008), 1.265 (6) and 1.275 (6) Å in [Mn(C_9_H_10_NO_2_)_2_(H_2_O)_4_]. 2(H_2_O), (III) (Hökelek & Necefoğlu, 2007), 1.260 (4) and 1.252 (4) Å in [Zn(DENA)_2_(C_7_H_4_FO_2_)_2_(H_2_O)_2_],(IV) (Hökelek *et al.*, 2007), 1.259 (9) and 1.273 (9) Å in Cu_2_(DENA)_2_(C_6_H_5_COO)_4_, (V) (Hökelek *et al.*, 1995), 1.279 (4) and 1.246 (4) Å in [Zn_2_(DENA)_2_(C_7_H_5_O_3_)_4_]. 2H_2_O, (VI) (Hökelek & Necefoğlu, 1996), 1.251 (6) and 1.254 (7) Å in [Co(DENA)_2_(C_7_H_5_O_3_)_2_(H_2_O)_2_], (VII) (Hökelek & Necefoğlu, 1997) and 1.278 (3) and 1.246 (3) Å in [Cu(DENA)_2_(C_7_H_4_NO_4_)_2_(H_2_O)_2_], (VIII) (Hökelek *et al.*, 1997). In (I), the average Zn—O bond length is 2.1261 (17) Å and the Zn atom is displaced out of the least-squares planes of the carboxylate groups (O1/C1/O2) and (O3/C8/O4) by -0.531 (1) Å and 0.023 (3) Å, respectively. The dihedral angle between the planar carboxylate groups and the adjacent benzene rings A (C2—C7) and B (C9—C14) are 4.87 (16)° and 2.23 (21)°, respectively, while those between rings A, B and C (N1/C15—C19) are A/B = 65.13 (8), A/C = 11.47 (7) and B/C = 74.83 (8) °. Intramolecular C—H···O hydrogen bond (Table 2) results in the formation of a six-membered ring D (Zn1/O1/O2/O6/C1/H61) adopting envelope conformation, with atom Zn1 displaced by 0.610 (1) Å from the plane of the other ring atoms. In (I), the O3—Zn1—O4 angle is 60.03 (6)°. The corresponding O—M—O (where *M* is a metal) angles are 58.3 (3)° in (VI) and 55.2 (1)° in [Cu(Asp)_2_(py)_2_] (where Asp is acetylsalicylate and py is pyridine) [(IX); Greenaway *et al.*, 1984].

In the crystal structure, strong intermolecular O—H···O, O—H···N and N—H···O hydrogen bonds (Table 2) link the molecules into a supramolecular structure, in which they may be effective in the stabilization of the structure. The π–π contacts between the pyridine and the benzene rings and the benzene rings, *Cg*1—*Cg*3^i^ and *Cg*2···*Cg*2^ii^ [symmetry codes: (i) *x* - 1, *y*, *z*, (ii) 1 - *x*, 1 - *y*, -*z*, where *Cg*1, *Cg*2 and *Cg*3 are centroids of the rings A (C2—C7), B (C9—C14) and C (N1/C15—C19), respectively] may further stabilize the structure, with centroid-centroid distances of 3.695 (1) and 3.841 (1) Å, respectively. There also exist two weak C—H···π interactions (Table 1).

### Experimental

The title compound was prepared by the reaction of ZnSO_4_.H_2_O (0.90 g, 5 mmol) in H_2_O (30 ml) and INA (1.22 g, 10 mmol) in H_2_O (20 ml) with sodium *p*-dimethylaminobenzoate (1.88 g, 10 mmol) in H_2_O (50 ml). The mixture was filtered and set aside to crystallize at ambient temperature for one week, giving colorless single crystals.

### Refinement

H atoms of water molecules and NH_2_ group were located in difference Fourier maps and refined isotropically, with restrains of O6—H61 = 0.891 (17), O6—H62 = 0.879 (19), O7—H71 = 0.89 (3), O7—H72 = 0.875 (18)Å and H61—O6—H62 = 106 (3) and H71—O7—H72 = 106 (2)°. The remaining H atoms were positioned geometrically with C—H = 0.93 and 0.96 Å, for aromatic and methyl H atoms, respectively, and constrained to ride on their parent atoms, with *U*_iso_(H) = *xU*_eq_(C), where *x* = 1.5 for methyl H and *x* = 1.2 for aromatic H atoms.

### Crystal data

**Table:**

[Zn(C_9_H_10_NO_2_)_2_(C_6_H_6_N_2_O)(H_2_O)_2_]	*Z* = 2
*M**_r_* = 551.91	*F*(000) = 576
Triclinic, *P*1	*D*_x_ = 1.471 Mg m^−^^3^
Hall symbol: -P 1	Mo *K*α radiation, λ = 0.71073 Å
*a* = 6.8616 (2) Å	Cell parameters from 9908 reflections
*b* = 8.0947 (3) Å	θ = 2.7–28.4°
*c* = 22.4953 (4) Å	µ = 1.04 mm^−^^1^
α = 90.683 (2)°	*T* = 100 K
β = 92.838 (2)°	Block, colorless
γ = 93.313 (3)°	0.57 × 0.27 × 0.20 mm
*V* = 1245.69 (6) Å^3^	

### Data collection

**Table:**

Bruker Kappa APEXII CCD area-detector diffractometer	6053 independent reflections
Radiation source: fine-focus sealed tube	5647 reflections with *I* > 2σ(*I*)
graphite	*R*_int_ = 0.021
φ and ω scans	θ_max_ = 28.4°, θ_min_ = 1.8°
Absorption correction: multi-scan (*SADABS*; Bruker, 2005)	*h* = −9→9
*T*_min_ = 0.720, *T*_max_ = 0.810	*k* = −10→10
21315 measured reflections	*l* = −30→30

### Refinement

**Table:**

Refinement on *F*^2^	Primary atom site location: structure-invariant direct methods
Least-squares matrix: full	Secondary atom site location: difference Fourier map
*R*[*F*^2^ > 2σ(*F*^2^)] = 0.040	Hydrogen site location: inferred from neighbouring sites
*wR*(*F*^2^) = 0.078	H atoms treated by a mixture of independent and constrained refinement
*S* = 1.29	*w* = 1/[σ^2^(*F*_o_^2^) + 1.9543*P*] where *P* = (*F*_o_^2^ + 2*F*_c_^2^)/3
6053 reflections	(Δ/σ)_max_ = 0.001
353 parameters	Δρ_max_ = 0.43 e Å^−^^3^
6 restraints	Δρ_min_ = −0.99 e Å^−^^3^

### Special details

**Table:**

Geometry. All e.s.d.'s (except the e.s.d. in the dihedral angle between two l.s. planes) are estimated using the full covariance matrix. The cell e.s.d.'s are taken into account individually in the estimation of e.s.d.'s in distances, angles and torsion angles; correlations between e.s.d.'s in cell parameters are only used when they are defined by crystal symmetry. An approximate (isotropic) treatment of cell e.s.d.'s is used for estimating e.s.d.'s involving l.s. planes.
Refinement. Refinement of *F*^2^ against ALL reflections. The weighted *R*-factor *wR* and goodness of fit *S* are based on *F*^2^, conventional *R*-factors *R* are based on *F*, with *F* set to zero for negative *F*^2^. The threshold expression of *F*^2^ > σ(*F*^2^) is used only for calculating *R*-factors(gt) *etc*. and is not relevant to the choice of reflections for refinement. *R*-factors based on *F*^2^ are statistically about twice as large as those based on *F*, and *R*- factors based on ALL data will be even larger.

### Fractional atomic coordinates and isotropic or equivalent isotropic displacement parameters (Å^2^)

**Table:**

	*x*	*y*	*z*	*U*_iso_*/*U*_eq_	
Zn1	0.43308 (4)	0.52665 (3)	0.277312 (12)	0.00968 (7)	
O1	0.6072 (2)	0.6802 (2)	0.33222 (7)	0.0139 (3)	
O2	0.8588 (2)	0.5241 (2)	0.35273 (8)	0.0151 (3)	
O3	0.3267 (2)	0.4011 (2)	0.19727 (7)	0.0136 (3)	
O4	0.6263 (2)	0.5140 (2)	0.19916 (7)	0.0140 (3)	
O5	−0.3021 (2)	1.0876 (2)	0.23256 (8)	0.0152 (3)	
O6	0.5716 (2)	0.3151 (2)	0.31154 (8)	0.0137 (3)	
H61	0.683 (3)	0.366 (4)	0.3259 (14)	0.034 (9)*	
H62	0.605 (5)	0.250 (4)	0.2826 (13)	0.043 (11)*	
O7	0.2121 (2)	0.4297 (2)	0.32786 (8)	0.0156 (4)	
H71	0.241 (5)	0.362 (4)	0.3573 (12)	0.041 (10)*	
H72	0.102 (4)	0.471 (4)	0.3366 (15)	0.040 (10)*	
N1	0.2604 (3)	0.7288 (2)	0.25405 (8)	0.0111 (4)	
N2	−0.0589 (3)	1.2657 (3)	0.20535 (10)	0.0150 (4)	
H21	−0.144 (5)	1.345 (4)	0.2033 (14)	0.028 (9)*	
H22	0.065 (5)	1.293 (4)	0.1978 (14)	0.026 (8)*	
N3	1.2354 (3)	1.2427 (2)	0.43495 (9)	0.0134 (4)	
N4	0.5670 (4)	0.1902 (3)	−0.06335 (10)	0.0295 (6)	
C1	0.7806 (3)	0.6619 (3)	0.35163 (10)	0.0118 (4)	
C2	0.8980 (3)	0.8119 (3)	0.37526 (10)	0.0116 (4)	
C3	1.0944 (3)	0.8040 (3)	0.39285 (10)	0.0133 (4)	
H3	1.1525	0.7032	0.3907	0.016*	
C4	1.2056 (3)	0.9437 (3)	0.41358 (10)	0.0137 (4)	
H4	1.3366	0.9350	0.4251	0.016*	
C5	1.1230 (3)	1.0971 (3)	0.41741 (10)	0.0116 (4)	
C6	0.9246 (3)	1.1047 (3)	0.39978 (11)	0.0148 (5)	
H6	0.8656	1.2049	0.4023	0.018*	
C7	0.8157 (3)	0.9653 (3)	0.37883 (10)	0.0138 (5)	
H7	0.6850	0.9738	0.3669	0.017*	
C8	0.4856 (3)	0.4329 (3)	0.17119 (10)	0.0118 (4)	
C9	0.5045 (4)	0.3732 (3)	0.10964 (10)	0.0138 (5)	
C10	0.3518 (4)	0.2825 (3)	0.07888 (11)	0.0186 (5)	
H10	0.2349	0.2609	0.0974	0.022*	
C11	0.3696 (4)	0.2240 (3)	0.02182 (12)	0.0223 (5)	
H11	0.2643	0.1656	0.0022	0.027*	
C12	0.5457 (4)	0.2517 (3)	−0.00729 (11)	0.0223 (6)	
C13	0.6994 (4)	0.3448 (3)	0.02368 (12)	0.0227 (6)	
H13	0.8169	0.3664	0.0056	0.027*	
C14	0.6774 (4)	0.4042 (3)	0.08049 (11)	0.0181 (5)	
H14	0.7802	0.4665	0.0999	0.022*	
C15	0.3272 (3)	0.8873 (3)	0.25996 (10)	0.0134 (5)	
H15	0.4576	0.9101	0.2721	0.016*	
C16	0.2101 (3)	1.0188 (3)	0.24869 (11)	0.0138 (5)	
H16	0.2620	1.1274	0.2524	0.017*	
C17	0.0147 (3)	0.9856 (3)	0.23173 (10)	0.0103 (4)	
C18	−0.0561 (3)	0.8214 (3)	0.22551 (10)	0.0117 (4)	
H18	−0.1868	0.7952	0.2145	0.014*	
C19	0.0719 (3)	0.6980 (3)	0.23603 (10)	0.0120 (4)	
H19	0.0254	0.5884	0.2304	0.014*	
C20	1.1361 (4)	1.3649 (3)	0.46894 (11)	0.0172 (5)	
H20A	1.2236	1.4601	0.4773	0.026*	
H20B	1.0964	1.3173	0.5056	0.026*	
H20C	1.0230	1.3975	0.4462	0.026*	
C21	1.4337 (4)	1.2219 (3)	0.45885 (13)	0.0240 (6)	
H21A	1.4981	1.3285	0.4675	0.036*	
H21B	1.5046	1.1645	0.4301	0.036*	
H21C	1.4290	1.1589	0.4947	0.036*	
C22	0.7510 (5)	0.2163 (4)	−0.09166 (13)	0.0376 (8)	
H22A	0.7477	0.1513	−0.1277	0.056*	
H22B	0.8556	0.1835	−0.0652	0.056*	
H22C	0.7719	0.3313	−0.1008	0.056*	
C23	0.4016 (5)	0.1097 (4)	−0.09693 (13)	0.0358 (8)	
H23A	0.4403	0.0798	−0.1359	0.054*	
H23B	0.2978	0.1841	−0.1005	0.054*	
H23C	0.3572	0.0119	−0.0767	0.054*	
C24	−0.1275 (3)	1.1191 (3)	0.22302 (10)	0.0117 (4)	

### Atomic displacement parameters (Å^2^)

**Table:**

	*U*^11^	*U*^22^	*U*^33^	*U*^12^	*U*^13^	*U*^23^
Zn1	0.00962 (12)	0.00784 (13)	0.01170 (12)	0.00169 (9)	0.00048 (9)	−0.00011 (9)
O1	0.0114 (8)	0.0130 (8)	0.0170 (8)	0.0028 (6)	−0.0028 (6)	−0.0015 (6)
O2	0.0125 (8)	0.0096 (8)	0.0232 (9)	0.0023 (6)	−0.0006 (7)	−0.0010 (7)
O3	0.0134 (8)	0.0131 (8)	0.0145 (8)	−0.0001 (6)	0.0033 (6)	−0.0011 (6)
O4	0.0138 (8)	0.0123 (8)	0.0156 (8)	−0.0018 (6)	−0.0003 (6)	−0.0008 (6)
O5	0.0109 (8)	0.0115 (8)	0.0232 (9)	0.0010 (6)	0.0018 (7)	−0.0018 (7)
O6	0.0108 (8)	0.0128 (9)	0.0177 (8)	0.0020 (6)	0.0005 (6)	0.0000 (7)
O7	0.0133 (8)	0.0166 (9)	0.0180 (8)	0.0055 (7)	0.0050 (7)	0.0059 (7)
N1	0.0113 (9)	0.0118 (10)	0.0102 (9)	−0.0001 (7)	0.0011 (7)	0.0004 (7)
N2	0.0107 (10)	0.0095 (10)	0.0249 (11)	0.0017 (8)	−0.0012 (8)	0.0026 (8)
N3	0.0125 (9)	0.0118 (10)	0.0155 (9)	0.0011 (7)	−0.0012 (7)	−0.0021 (8)
N4	0.0458 (16)	0.0308 (14)	0.0131 (11)	0.0092 (12)	0.0045 (10)	−0.0039 (9)
C1	0.0141 (11)	0.0119 (11)	0.0096 (10)	0.0010 (8)	0.0016 (8)	−0.0002 (8)
C2	0.0127 (11)	0.0122 (11)	0.0100 (10)	0.0003 (8)	0.0011 (8)	−0.0001 (8)
C3	0.0140 (11)	0.0104 (11)	0.0157 (11)	0.0027 (8)	0.0004 (9)	0.0006 (9)
C4	0.0115 (11)	0.0148 (12)	0.0148 (11)	0.0022 (9)	−0.0014 (8)	0.0011 (9)
C5	0.0140 (11)	0.0111 (11)	0.0097 (10)	0.0005 (8)	0.0004 (8)	0.0007 (8)
C6	0.0143 (11)	0.0114 (11)	0.0189 (11)	0.0044 (9)	−0.0005 (9)	−0.0010 (9)
C7	0.0114 (11)	0.0143 (12)	0.0156 (11)	0.0024 (9)	−0.0009 (8)	−0.0008 (9)
C8	0.0120 (11)	0.0088 (11)	0.0151 (11)	0.0027 (8)	0.0007 (8)	0.0017 (8)
C9	0.0170 (11)	0.0120 (11)	0.0126 (11)	0.0027 (9)	0.0009 (9)	0.0007 (8)
C10	0.0202 (13)	0.0200 (13)	0.0154 (12)	0.0008 (10)	0.0003 (9)	−0.0016 (10)
C11	0.0285 (14)	0.0203 (14)	0.0176 (12)	0.0024 (11)	−0.0044 (10)	−0.0039 (10)
C12	0.0370 (16)	0.0181 (13)	0.0128 (11)	0.0090 (11)	0.0020 (10)	0.0007 (10)
C13	0.0274 (14)	0.0234 (14)	0.0186 (12)	0.0053 (11)	0.0093 (10)	0.0029 (10)
C14	0.0213 (13)	0.0170 (13)	0.0162 (11)	0.0013 (10)	0.0035 (9)	0.0008 (9)
C15	0.0113 (11)	0.0123 (11)	0.0166 (11)	−0.0003 (8)	−0.0003 (8)	0.0031 (9)
C16	0.0128 (11)	0.0094 (11)	0.0190 (11)	−0.0002 (8)	0.0004 (9)	0.0009 (9)
C17	0.0113 (10)	0.0097 (11)	0.0103 (10)	0.0021 (8)	0.0018 (8)	0.0004 (8)
C18	0.0102 (10)	0.0116 (11)	0.0131 (10)	0.0008 (8)	0.0006 (8)	−0.0004 (8)
C19	0.0141 (11)	0.0079 (11)	0.0136 (10)	−0.0015 (8)	−0.0001 (8)	−0.0009 (8)
C20	0.0220 (13)	0.0149 (12)	0.0148 (11)	0.0026 (9)	0.0013 (9)	−0.0029 (9)
C21	0.0171 (13)	0.0168 (13)	0.0372 (16)	0.0029 (10)	−0.0089 (11)	−0.0057 (11)
C22	0.056 (2)	0.043 (2)	0.0170 (14)	0.0158 (16)	0.0132 (14)	−0.0017 (13)
C23	0.057 (2)	0.0350 (18)	0.0164 (13)	0.0177 (16)	−0.0076 (13)	−0.0072 (12)
C24	0.0120 (11)	0.0103 (11)	0.0127 (10)	0.0013 (8)	−0.0004 (8)	−0.0023 (8)

### Geometric parameters (Å, °)

**Table:**

Zn1—O1	2.0353 (17)	C7—C6	1.381 (3)
Zn1—O3	2.1328 (17)	C7—H7	0.9300
Zn1—O4	2.2584 (17)	C9—C8	1.475 (3)
Zn1—O6	2.1393 (17)	C9—C10	1.394 (3)
Zn1—O7	2.0647 (17)	C9—C14	1.395 (3)
Zn1—N1	2.1307 (19)	C10—C11	1.376 (4)
Zn1—C8	2.545 (2)	C10—H10	0.9300
O1—C1	1.265 (3)	C11—H11	0.9300
O2—C1	1.265 (3)	C12—C11	1.410 (4)
O3—C8	1.278 (3)	C13—C12	1.410 (4)
O4—C8	1.271 (3)	C13—H13	0.9300
O5—C24	1.240 (3)	C14—C13	1.378 (4)
O6—H61	0.891 (17)	C14—H14	0.9300
O6—H62	0.879 (19)	C15—C16	1.389 (3)
O7—H71	0.89 (3)	C15—H15	0.9300
O7—H72	0.875 (18)	C16—C17	1.386 (3)
N1—C15	1.340 (3)	C16—H16	0.9300
N1—C19	1.345 (3)	C18—C17	1.392 (3)
N2—H21	0.89 (3)	C18—C19	1.382 (3)
N2—H22	0.89 (3)	C18—H18	0.9300
N3—C5	1.412 (3)	C19—H19	0.9300
N3—C20	1.463 (3)	C20—H20A	0.9600
N3—C21	1.458 (3)	C20—H20B	0.9600
N4—C12	1.368 (3)	C20—H20C	0.9600
N4—C22	1.448 (4)	C21—H21A	0.9600
N4—C23	1.449 (4)	C21—H21B	0.9600
C1—C2	1.495 (3)	C21—H21C	0.9600
C3—C2	1.391 (3)	C22—H22A	0.9600
C3—C4	1.389 (3)	C22—H22B	0.9600
C3—H3	0.9300	C22—H22C	0.9600
C4—C5	1.398 (3)	C23—H23A	0.9600
C4—H4	0.9300	C23—H23B	0.9600
C5—C6	1.404 (3)	C23—H23C	0.9600
C6—H6	0.9300	C24—N2	1.325 (3)
C7—C2	1.396 (3)	C24—C17	1.505 (3)
			
O1—Zn1—O3	158.68 (7)	C10—C9—C8	121.4 (2)
O1—Zn1—O4	99.43 (6)	C10—C9—C14	117.7 (2)
O1—Zn1—O6	91.08 (7)	C14—C9—C8	120.9 (2)
O1—Zn1—O7	106.57 (7)	C9—C10—H10	119.2
O1—Zn1—N1	89.59 (7)	C11—C10—C9	121.6 (2)
O3—Zn1—O4	60.03 (6)	C11—C10—H10	119.2
O3—Zn1—O6	93.40 (7)	C10—C11—C12	120.8 (3)
O6—Zn1—O4	87.44 (6)	C10—C11—H11	119.6
O7—Zn1—O3	94.72 (7)	C12—C11—H11	119.6
O7—Zn1—O4	151.55 (7)	N4—C12—C11	121.4 (3)
O7—Zn1—O6	80.80 (7)	N4—C12—C13	121.2 (3)
O7—Zn1—N1	89.90 (7)	C11—C12—C13	117.4 (2)
N1—Zn1—O3	89.40 (7)	C12—C13—H13	119.6
N1—Zn1—O4	101.82 (7)	C14—C13—C12	120.8 (2)
N1—Zn1—O6	170.47 (7)	C14—C13—H13	119.6
C1—O1—Zn1	128.40 (15)	C9—C14—H14	119.2
C8—O3—Zn1	93.10 (14)	C13—C14—C9	121.6 (3)
C8—O4—Zn1	87.63 (13)	C13—C14—H14	119.2
Zn1—O6—H61	98 (2)	N1—C15—C16	122.8 (2)
Zn1—O6—H62	111 (2)	N1—C15—H15	118.6
H61—O6—H62	106 (3)	C16—C15—H15	118.6
Zn1—O7—H71	119 (2)	C15—C16—H16	120.6
Zn1—O7—H72	130 (2)	C17—C16—C15	118.9 (2)
H72—O7—H71	106 (2)	C17—C16—H16	120.6
C15—N1—Zn1	123.02 (16)	C16—C17—C18	118.7 (2)
C15—N1—C19	117.8 (2)	C16—C17—C24	122.9 (2)
C19—N1—Zn1	119.03 (16)	C18—C17—C24	118.3 (2)
C24—N2—H21	116 (2)	C17—C18—H18	120.7
C24—N2—H22	126 (2)	C19—C18—C17	118.6 (2)
H22—N2—H21	118 (3)	C19—C18—H18	120.7
C5—N3—C21	116.7 (2)	N1—C19—C18	123.1 (2)
C5—N3—C20	116.3 (2)	N1—C19—H19	118.4
C21—N3—C20	112.2 (2)	C18—C19—H19	118.4
C12—N4—C22	120.3 (3)	N3—C20—H20A	109.5
C12—N4—C23	120.4 (3)	N3—C20—H20B	109.5
C22—N4—C23	119.1 (2)	N3—C20—H20C	109.5
O1—C1—O2	123.7 (2)	H20A—C20—H20B	109.5
O1—C1—C2	117.7 (2)	H20A—C20—H20C	109.5
O2—C1—C2	118.5 (2)	H20B—C20—H20C	109.5
C3—C2—C1	121.0 (2)	N3—C21—H21A	109.5
C3—C2—C7	117.9 (2)	N3—C21—H21B	109.5
C7—C2—C1	121.2 (2)	N3—C21—H21C	109.5
C2—C3—H3	119.4	H21A—C21—H21B	109.5
C4—C3—C2	121.3 (2)	H21A—C21—H21C	109.5
C4—C3—H3	119.4	H21B—C21—H21C	109.5
C3—C4—C5	120.9 (2)	N4—C22—H22A	109.5
C3—C4—H4	119.6	N4—C22—H22B	109.5
C5—C4—H4	119.6	N4—C22—H22C	109.5
C4—C5—N3	122.0 (2)	H22A—C22—H22B	109.5
C4—C5—C6	117.7 (2)	H22A—C22—H22C	109.5
C6—C5—N3	120.2 (2)	H22B—C22—H22C	109.5
C5—C6—H6	119.5	N4—C23—H23A	109.5
C7—C6—C5	121.0 (2)	N4—C23—H23B	109.5
C7—C6—H6	119.5	N4—C23—H23C	109.5
C2—C7—H7	119.3	H23A—C23—H23B	109.5
C6—C7—C2	121.3 (2)	H23A—C23—H23C	109.5
C6—C7—H7	119.3	H23B—C23—H23C	109.5
O3—C8—C9	119.8 (2)	O5—C24—N2	123.0 (2)
O4—C8—O3	119.2 (2)	O5—C24—C17	119.1 (2)
O4—C8—C9	121.0 (2)	N2—C24—C17	117.9 (2)
			
O3—Zn1—O1—C1	−71.5 (3)	C23—N4—C12—C13	−173.5 (3)
O4—Zn1—O1—C1	−56.9 (2)	O1—C1—C2—C3	−174.7 (2)
O6—Zn1—O1—C1	30.69 (19)	O1—C1—C2—C7	3.5 (3)
O7—Zn1—O1—C1	111.42 (19)	O2—C1—C2—C3	6.0 (3)
N1—Zn1—O1—C1	−158.81 (19)	O2—C1—C2—C7	−175.8 (2)
O1—Zn1—O3—C8	16.4 (2)	C4—C3—C2—C1	178.5 (2)
O4—Zn1—O3—C8	−0.36 (12)	C4—C3—C2—C7	0.3 (3)
O6—Zn1—O3—C8	−85.43 (13)	C2—C3—C4—C5	0.0 (4)
O7—Zn1—O3—C8	−166.47 (13)	C3—C4—C5—N3	−176.2 (2)
N1—Zn1—O3—C8	103.68 (13)	C3—C4—C5—C6	0.2 (3)
O1—Zn1—O4—C8	−173.56 (13)	N3—C5—C6—C7	175.8 (2)
O3—Zn1—O4—C8	0.36 (12)	C4—C5—C6—C7	−0.7 (3)
O6—Zn1—O4—C8	95.77 (13)	C6—C7—C2—C1	−179.0 (2)
O7—Zn1—O4—C8	30.5 (2)	C6—C7—C2—C3	−0.8 (3)
N1—Zn1—O4—C8	−81.99 (13)	C2—C7—C6—C5	1.0 (4)
O1—Zn1—N1—C15	22.18 (18)	C10—C9—C8—O3	0.2 (3)
O1—Zn1—N1—C19	−153.57 (17)	C10—C9—C8—O4	179.2 (2)
O3—Zn1—N1—C15	−136.52 (18)	C14—C9—C8—O3	−179.1 (2)
O3—Zn1—N1—C19	47.73 (17)	C14—C9—C8—O4	−0.1 (3)
O4—Zn1—N1—C19	106.89 (17)	C8—C9—C10—C11	−178.9 (2)
O4—Zn1—N1—C15	−77.36 (18)	C14—C9—C10—C11	0.4 (4)
O7—Zn1—N1—C15	128.76 (18)	C8—C9—C14—C13	178.0 (2)
O7—Zn1—N1—C19	−46.99 (17)	C10—C9—C14—C13	−1.3 (4)
Zn1—O1—C1—O2	−19.5 (3)	C9—C10—C11—C12	1.1 (4)
Zn1—O1—C1—C2	161.36 (15)	N4—C12—C11—C10	178.1 (3)
Zn1—O3—C8—O4	0.6 (2)	C13—C12—C11—C10	−1.7 (4)
Zn1—O3—C8—C9	179.65 (18)	C14—C13—C12—N4	−179.0 (2)
Zn1—O4—C8—O3	−0.6 (2)	C14—C13—C12—C11	0.8 (4)
Zn1—O4—C8—C9	−179.61 (19)	C9—C14—C13—C12	0.8 (4)
Zn1—N1—C15—C16	−175.38 (17)	N1—C15—C16—C17	1.3 (4)
C19—N1—C15—C16	0.4 (3)	C15—C16—C17—C18	−1.2 (3)
Zn1—N1—C19—C18	173.64 (17)	C15—C16—C17—C24	175.2 (2)
C15—N1—C19—C18	−2.3 (3)	C19—C18—C17—C16	−0.5 (3)
C20—N3—C5—C4	−146.3 (2)	C19—C18—C17—C24	−177.2 (2)
C20—N3—C5—C6	37.4 (3)	C17—C18—C19—N1	2.4 (3)
C21—N3—C5—C4	−10.4 (3)	O5—C24—C17—C16	−150.0 (2)
C21—N3—C5—C6	173.4 (2)	O5—C24—C17—C18	26.5 (3)
C22—N4—C12—C11	−178.3 (3)	N2—C24—C17—C16	30.0 (3)
C22—N4—C12—C13	1.5 (4)	N2—C24—C17—C18	−153.5 (2)
C23—N4—C12—C11	6.7 (4)		

### Hydrogen-bond geometry (Å, °)

**Table:**

*D*—H···*A*	*D*—H	H···*A*	*D*···*A*	*D*—H···*A*
N2—H21···O4^i^	0.89 (3)	2.15 (3)	3.034 (3)	174 (3)
N2—H22···O3^ii^	0.89 (3)	1.95 (3)	2.820 (3)	164 (3)
O6—H61···O2	0.89 (3)	1.79 (3)	2.646 (2)	161 (3)
O6—H62···O5^iii^	0.88 (3)	1.88 (3)	2.749 (2)	169 (3)
O7—H71···N3^iv^	0.89 (3)	2.01 (3)	2.863 (3)	162 (3)
O7—H72···O2^v^	0.87 (3)	1.80 (3)	2.667 (2)	171 (3)
C22—H22A···Cg3^vi^	0.96	2.74	3.567 (3)	145
C23—H23C···Cg2^vii^	0.96	2.86	3.644 (3)	140

Symmetry codes: (i) *x*−1, *y*+1, *z*; (ii) *x*, *y*+1, *z*; (iii) *x*+1, *y*−1, *z*; (iv) *x*−1, *y*−1, *z*; (v) *x*−1, *y*, *z*; (vi) −*x*+1, −*y*+1, −*z*; (vii) −*x*+1, −*y*, −*z*.
